# Folding photopolymerized origami sheets by post-curing

**DOI:** 10.1007/s42452-020-04018-w

**Published:** 2021-01-13

**Authors:** Xiaodong He, Christopher-Denny Matte, Tsz-Ho Kwok

**Affiliations:** Department of Mechanical, Industrial and Aerospace Engineering, Concordia University, Montreal, Canada

**Keywords:** Elastomer, Digital light processing, Post-curing, Hinge design, Origami

## Abstract

The paper presents a novel manufacturing approach to fabricate origami based on 3D printing utilizing digital light processing. Specifically, we propose to leave part of the model uncured during the printing step, and then cure it in the post-processing step to set the shape in a folded configuration. While the cured regions in the first step try to regain their unfolded shape, the regions cured in the second step attempt to keep their folded shape. As a result, the final shape is obtained when both regions’ stresses reach equilibrium. Finite element analysis is performed in ANSYS to obtain the stress distribution on common hinge designs, demonstrating that the square-hinge has a lower maximum principal stress than elliptical and triangle hinges. Based on the square-hinge and rectangular cavity, two variables—the hinge width and the cavity height—are selected as principal variables to construct an empirical model with the final folding angle. In the end, experimental verification shows that the developed method is valid and reliable to realize the proposed deformation and 3D development of 2D hinges.

## Introduction

Origami, stemming from ancient Japanese art, has gained popularity in the past centuries because of its ability to create complex three-dimensional (3D) shapes from a two-dimensional (2D) sheet of paper strictly using folding techniques. Origami has been applied in many engineering applications to build up 3D shapes [32], such as protective articles [26], aerospace [14], communication equipment [29], and medical devices [8]. In these cases, engineering materials with non-negligible thickness are applied to have the necessary stiffness to perform different functions. However, to fold the materials with non-negligible thickness and hold the final shape, certain assembly steps or mechanical fasteners are needed. Otherwise, it is limited to the materials with plastic deformation (e.g., ductile metal and paper through delamination of layers). Recently, there are various self-folding mechanisms proposed for the fabrication of origami-based products. For example, smart materials like shape-memory polymers or alloys are applied to deform shapes in a pre-programmed manner [11]. Additionally, concepts such as four-dimensional (4D) printing, achieved through selective material placement during 3D printing [20], allow for new self-folding mechanism through non-homogeneous deformation by combining materials with different expansion ratios [22].

The self-folding mechanisms require the use of either smart materials or multiple materials. Some examples include origami patterns using NiTi coils as actuators [27], heated pre-programmed polymers [12], self-assembling microfluidics [13], or using electric fields to actuate P-Terpolymers  [1]. However, the complexity of multi-material fabrication, material cost, and production time hinder a wider use of origami structures in engineering applications. The interesting question here is: *how to expend the use of ‘non-smart’ materials in origami, such that they can be fabricated in 2D and folded to their final 3D shapes?* The question motivates our research to develop a user-friendly way to apply non-smart materials to origami-based products without increasing the manufacturing process’s complexity. Since the manufactured part needs to be deformed to its final shape, this paper primarily focuses on elastic materials.

To address the research question, we revisited the 3D printing technologies to search for possibilities. We observed that many 3D printing processes need a post-processing step. For example, fused deposition method (FDM) applies vaporization  [3] or coating [41] after printing to improve the surface finishing; the selective laser sintering (SLS) method applies a post-sintering step to increase density and enhance the mechanical strength of the printed parts [38]; the stereolithography (SLA) or digital light processing (DLP) technology has a post-curing step to maximize the material’s physical properties.

With this observation, we hypothesize that deforming a 3D-printed part in the post-processing step will enable it to keep the final deformed shape, even after removing all external stimuli. If tested true, we will be able to use the post-processing step to realize the folding to final shapes for non-smart materials. Previous works [29, 33] investigate 3D printed resins’ over-curing properties to instill continuous bending in specific sections. By controlling the light intensity and exposure during a print, the process enables a single material to have multiple behaviors. However, this approach is limited to thin structures with minimal structural strength and cannot create sharp bends at desired locations. To test this hypothesis and study the extent of its ability to keep the deformed shape, we pick the DLP 3D printing technology for a proof-of-concept, study the principle of its post-curing operation, and develop the design and manufacturing methodology to allow shape-retention. DLP 3D printing takes advantage of a digital light projector to cure a photopolymer resin layer-by-layer. The post-curing is similar to a second solidification step after the initial 3D printing. Our goal is to use this two-step curing process to introduce different stresses within the model such that it will retain its 3D shape. Specifically, we propose to leave part of the model uncured during the printing step and then cure it in the post-processing curing step after folding. In this way, while the regions cured in the first step tend to go back to the unfolded shape, the regions cured in the second step try to keep its folded shape. As a result, the final shape is obtainable by the balance between them, i.e., at the stresses’ equilibrium. The contributions of the paper comes from developing and testing such a methodology, which are summarized as follows.

- A new method is developed to fabricate 3D shapes from 2D geometry, requiring no assembly and without changing the DLP 3D printing manufacturing pipeline.
- Various geometries are studied, and a new hinge design is developed to trap the uncured material during 3D printing and allow folding without leakage.
- To enable the control of the desired deformation, a mathematical relationship between the folding angle and the hinge geometry is obtained experimentally.

Several origami examples are fabricated to demonstrate the proposed method’s capability, and the experimental results verify that the parts fabricated by 2D elastic material can hold the shapes in 3D and possess the origami characteristics.

The rest of the paper is organized as follows. In Sect. 2, a brief overview of current research is presented. The technological background and experimental setup, as well as the material characterization, are introduced in Sect. 3. In Sect. 4, the framework of the methodology is stated in detail, including the hinge design and the mathematical model for the folding angle. In Sect. 5, some testing cases with a few demonstrated uses of the method are presented, and the paper is concluded in Sect. 6.

## Literature review

This paper is related to origami fabrication, 3D printing and SLA/DLP with post-curing, the related works are presented below respectively.

### Origami modeling and fabrication

There is enormous interest in origami, and many works have been done on modeling and simulating paper origami. Several structures have been developed, such as the Miura-ori structuret [32] and the developable waterbomb tesselation  [40]. Additionally, the position and trajectories of coupled spherical joints have been derived for simulation [6]. Tachi [35] proposed a geometric simulation method for folding a generic rigid origami. The mechanical loading capabilities of an origami system can also be efficiently simulated [21, 42]. However, special considerations, such as the origami’s material properties, need to be placed when surfaces are not perfectly rigid compared to the joints. Saito et al. [30] proposed a mathematical model to evaluate the elastic deformation of non-rigid origami structures and demonstrated through deployable plate models. Fuchi et al. [10] put forward an optimization method combining finite-element analysis to distribute the mechanical properties within a flat structure. Similarly, simulation origami with non-negligible width in the creases was developped [28]. Bowen et al. [7] established a dynamic model of a waterbomb structure to approximate the torque produced by magneto-active elastomers when applied to the self-folding of waterbomb. Origami also has many engineering applications such as origami-inspired antennas  [5, 29] and medical forceps  [8, 23]. Additionally, an integrated fabrication framework of robotic application for building origami system in cm-scale  [4] based on the research of Felton [9] is possible. Previous research, such as capillary self-folding, has also utilized liquid and hybrid hinges to develop folding structures [19]. Although capillary forces are ideal for nano and micro-scale applications $(<1N)$, the force output is negligible on the macro scale.

### 3D printing origami

Fabricating origami is complex, and more efforts are using 3D printing for manufacturing origami products. Several works have utilized inkjet printing of hinges to control folding angles and generate complex forms  [16, 39] Ahn et al. [2] used diverse materials and combined direct-write and wet folding techniques to manufacture origami, with shapes ranging from simple polyhedra to intricate origami forms. Wu et al. [34] presented an approach to print reconfigurable antennas by combining Liquid Metal Alloy (LMA) microfluidics and Voronoi origami structures. Mao et al. [24] printed sequential self-folding structures by thermal activation of spatially-variable patterns with digital shape memory polymers. Ge et al. [11] printed active composite by the PolyJet technology, in which shape memory fibers are embedded in an elastomeric matrix at the hinges to enable origami folding. However, all these methods either require smart materials or an accurate external actuation is needed to fold the material.

## Technological background and material

### Digital light processing 3D printing

Digital light processing (DLP) is a type of vat polymerization, which utilizes a photopolymer resin and a digital light projector as the light source to produce parts. A photopolymer is a photo-reactive polymer that cures (or solidifies) when exposed to light. Therefore, by sequentially projecting different mask images (black and white) layer-by-layer, different cross-sectional areas are fabricated, and a 3D part is produced.

A typical processing sequence to fabricate a discrete part consists of shaping processes, property-enhancing operations, and finishing operations. Similarly, the layer-by-layer projection is the shaping process in the DLP 3D printing, and is followed by a post-curing process for enhancing the property of the part. During the shaping process, each layer’s exposure time is calibrated and optimized, such that there is no under-curing or over-curing to maintain dimensional accuracy. However, the material is not completely cured during the shaping process. Therefore, after the whole part is shaped, it is soaked in an ultrasonic cleaner with $99\%$ isopropyl alcohol for three minutes to wash away the residual resin on the surface. Then it undergoes the post-curing step for ten minutes to finish and enhance the mechanical properties of parts fabricated by SLA/DLP printing. The post-curing step is often necessary and is especially important for functional resin [17, 37].

Several studies have been performed to understand the mechanical effects of post curing [17, 31], from characterizing curing strategies [25], investigating the use of epoxy additives [18], to the influence of photo-initiators  [33]. Wu et al. [37] studied the influence of post-curing on shape integrity and dimension accuracy and characterized the evolution of samples’ mechanical behavior during post-curing. Dimensional accuracy, surface roughness, building orientation, and mechanical properties of printed structures based on the glass transition temperature of the resin system under UV-curing has also been investigated  [15].

### Experimental setup and material

**Fig. 1:**
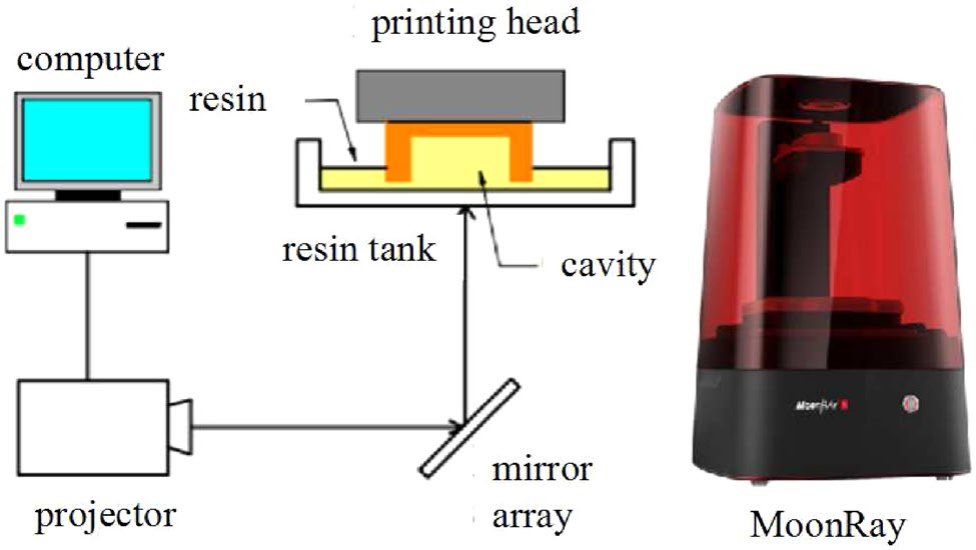
Mechanism of the DLP printing process and MoonRay printer

**Table 1:** Material properties of the photo-polymer used in this study

Material parameter	Value
Density	1100 (${\mathrm{kg}}/{\mathrm{m}}^3)$
Viscosity at 77 $^{\circ }$F/25 $^{\circ }$C	1300–1500 (cP)
Hardness (Shore)	82–85 (A)
Ultimate strength	0.84 (MPa)
Elongation at break	140 (%)

**Fig. 2:**
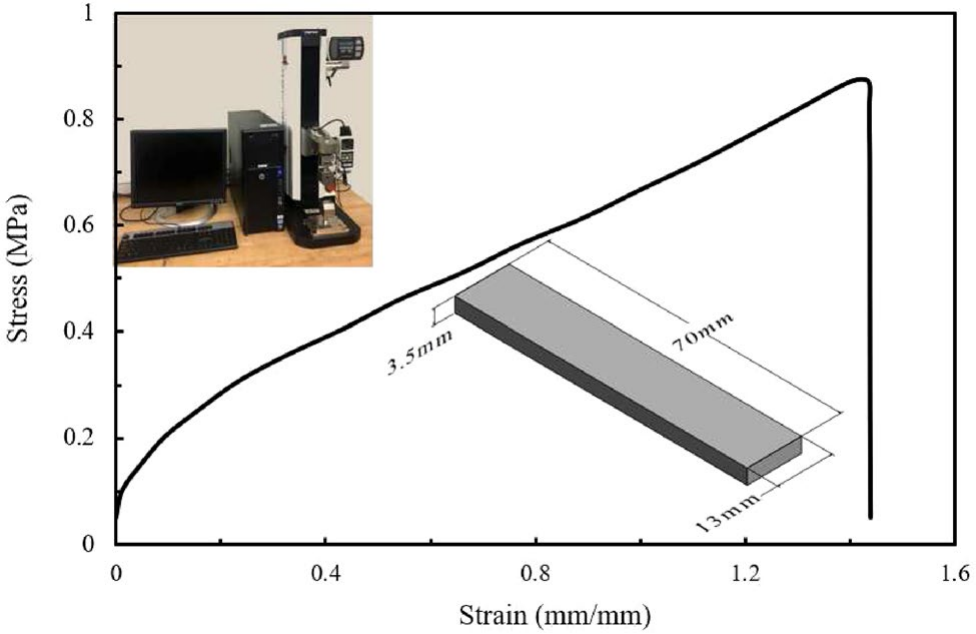
Stress-stain curve of the material used and experiment setting

In this paper, the MoonRay S100 DLP 3D printer produced by SprintRay is utilized. It uses the bottom-up approach, as illustrated in Fig. 1. The printing volume of the machine is 130 mm $\times$ 80 mm $\times$ 200 mm. The ultrasonic cleaner SS65 produced by Crystal Electronics, Inc. (Newmarket, ON) is used to remove the residual resin on the shaped part and the LC-3DPrint Box produced by 3D Systems, Inc. (Rock Hill, SC) is employed for post-curing the material.

The resin used is the PT-F001MT Prototype Flex photopolymer produced by ApplyLabWork (Torrance, CA), with its property presented in Table 1.

We are using a different manufacturing method than the producer’s to fabricate the material; thus, a tensile test obtains the mechanical properties of the material in this paper. Following the ASTM Standard Test Method D368, rectangular samples with the sizes of 70 mm $\times$ 13 mm $\times$ 3.5 mm are fabricated with the DLP 3D printer.

The testing is repeated ten times on the ESM750S motorized test stand produced by Mark-10 Corporation (Copiague, NY). To ensure reliability and reproducibility of our method, the group with the lowest stress and strain is retained and shown in Fig. 2. From the graph, the maximum stress and strain are 0.87 MPa and 1.4, respectively.

## Methodology

**Fig. 3:**
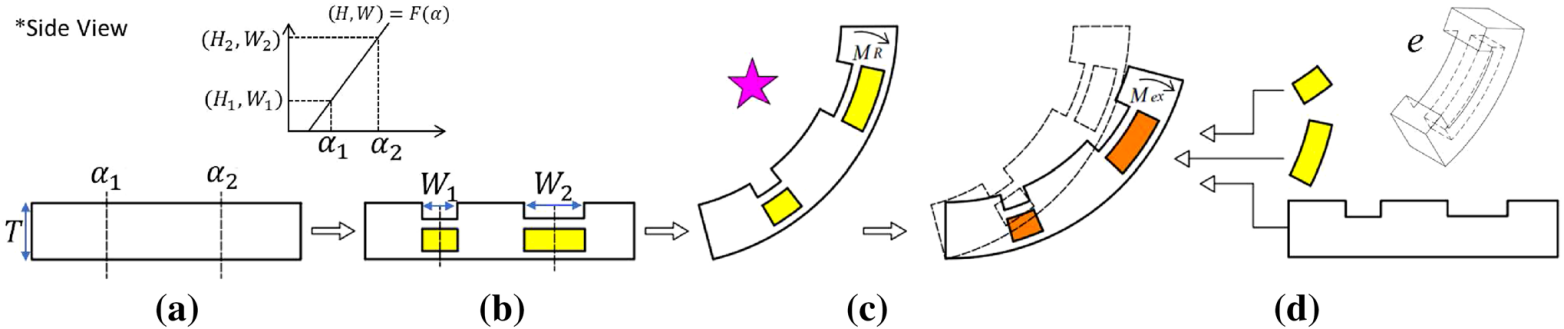
An illustration of the proposed process. **a** Initial origami pattern with two hinges of bending angles $\alpha _1$ and $\alpha _2$. **b** Through a calibrated mathematical model, the hinge parameters are obtained and the hinges are designed accordingly with cavity to contain uncured resin. **c** External moment is applied to deform the material, and post-curing is conducted to cure the resin inside the cavity. **d** The shape is maintained in folded shape even after the external moment is removed, as a balance between the stresses of the material cured in different steps. **e** An isometric view of a hinge with cavity

To obtain 3D shapes from the fabrication of 2D sheets of ‘non-smart’ materials, our approach leaves a part of the model uncured during the printing process and utilizes the post-curing process to set its 3D shape. To realize this approach, the 2D sheets must be designed with the capabilities to:

1. Trap the resin inside the model during the printing process.
2. Contain the resin in place without leakage during deformation.
3. Resist deformation after post-curing.

Since our work is motivated by origami where deformation occurs only at the fold lines, research was performed on the hinge design. Referring to the illustration shown in Fig. 3, given an origami pattern with defined fold lines and angles ($\alpha$), the fold lines are converted to 3D hinges with internal cavities of height *H* and widths *W*. We propose to include a cavity (in yellow) for every hinge such that it contains uncured resin after the 2D sheet is fabricated. The hinges’ design facilitates folding in a particular direction, similarly to creating creases in a paper before folding. In this case, they are directly 3D-printed, meaning there is no need to manually and individually crease each fold to obtain the final shape. An overall actuation can then automatically fold all hinges. A post-curing process is done once the 2D sheet is folded to fully cure the material and the uncured resin inside the cavity. After the resin is cured (in orange), it will try to retain its folded shape, and thus the final 3D shape will be obtained.

Mathematically speaking, when an external actuation folds the hinge (Fig. 3c), it has an internal reaction moment $M_R$. After the resin in the cavity is cured (Fig. 3d), the external actuation is released. The reaction moment $M_R$ then acts as the external load $M_{ex}$ to deform the newly cured material, which in turn generates an internal reaction moment $M_{in}$ in the cavity region, until they balance out each other and reach equilibrium, i.e., $M_{ex} = M_{in}$. Therefore there are two methods to retain the actuated state as much as possible after post-curing. One way is to maximize the moment $M_{in}$ in the cavity region, and the other way is to minimize the moment $M_R$ in the rest of the part. In Sect.  4.1 the design and development to address all the requirements mentioned above are presented.

### Hinge design

A cavity is introduced into each hinge design to trap uncured resin in the model. The cavity is fabricated as shown in Fig. 1. It is important that the cavity can retain the resin inside during the shaping process. That is, the cavity cannot be raised higher than the resin level during printing, and the weight of the resin inside the cavity should be small enough so that the atmospheric pressure can keep them in place. Similar to the principle used in barometers, the pressure in the cavity increases with height. It can be calculated as:

1 $$\begin{aligned} P=\rho gh, \end{aligned}$$

where *P* is the atmospheric pressure, $\rho$ is the density of resin, *g* is the gravity constant, and *h* is the height of the cavity above the resin level.

Given *P*=101,325 Pa, $\rho =1100\,{\mathrm{kg}}/{\mathrm{m}}^3$, and $g=9.8\,{\mathrm{m}}/{\mathrm{s}}^2$, the maximum height of cavity that can keep the uncured resin in place is 9.4 m. Since it is far more extensive than the typical sizes fabricated using DLP 3D printing, this limiting factor would never be reached. However, this does not validate the method entirely as the bottom surface of the cavity must also retain the resin inside. From various tests during this method’s calibration, a cavity height of over 10 mm was achieved with no issues. As such, for the range of DLP origami being 2–4 mm, the material and process are acceptable. The absolute limits of the cavity height are not presented and would be dependent on the material selected.

**Fig. 4:**
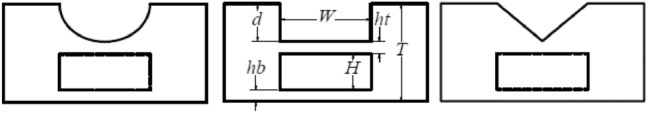
The sectional schematic of three hinges: elliptical, square, and triangle. *T*-thickness of the material. *H*-height of the cavity. *hb*-thickness below the cavity. *ht*-thickness above the cavity. *W*-width of the hinge. *d*-depth of the hinge notch

**Fig. 5:**
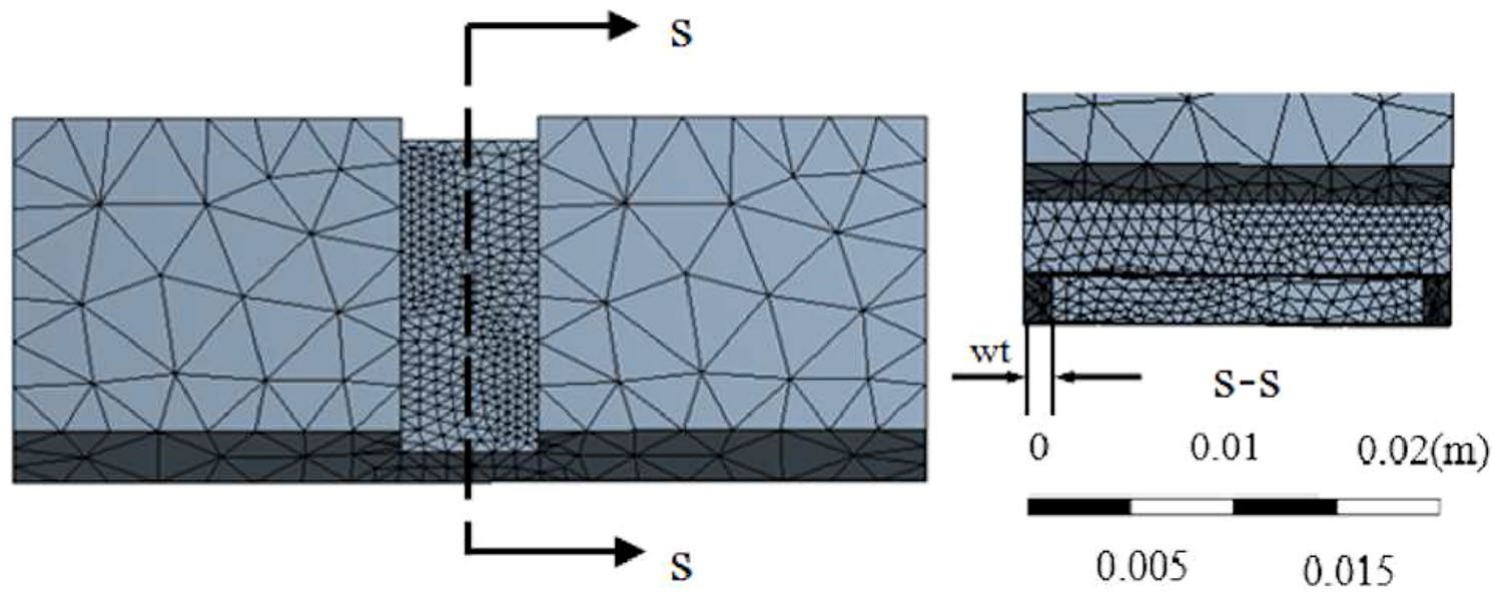
Mesh of square hinge used for simulation

Flexure hinges have been used to replace other bearings and universal joints because they are simple, compact, lightweight, and have low friction. Different designs are applied in medical, micro, and nanoscale applications, in which elliptical, square, and triangle hinges are the most common geometries [36] (see Fig. 4). From these three geometries, the goal is to determine the hinge that can reach the largest folding angle without leakage of resin from the cavity during folding. Minimizing the reaction moment $M_R$ maximizes the folding angle. Thus, it is needed to minimize the material experiencing the initial deformation and maximize the volume of the cavity. Referring to Figs. 4 and 5, besides the thickness of the hinge (*T*) that is given as an input, there are five other parameters. They are listed as follows: the opening of the hinge (*W*); the height of cavity (*H*); the top and bottom walls of cavity (*ht*, *hb*); and the sidewalls of the cavity (*wt*). The parameters *hb* and *ht* are dependent on *H* and should be minimized. *wt* is another parameter the intuitively should be minimized to allow for a larger cavity. However, to prevent leakage and maintain structural integrity, the maximum stress should not be higher than the ultimate stress throughout the process. In other words, the hinge design’s objective is to minimize the material around the hinge and maximize cavity size, thus minimizing the reaction moment. However, the cavity walls’ maximum stress still needs to be less than the material’s ultimate strength.

To find out which geometry has the lowest maximum stress during folding, models of the three shapes are built and simulated in ANSYS. The model’s length, width, and height are 40 mm, 15 mm, and 5.5 mm, respectively. The thickness of the 2D sheet is $T=5.5\,{\mathrm{mm}}$ and the depth of the hinge is $d=2.3\,{\mathrm{mm}}$. A tetrahedral mesh is used, and the subdivision at the hinge area is doubled to achieve higher accuracy (see Fig. 5). There are in total about 10*k* elements and 7k nodes in the simulation mesh. One end of the model is fixed, and a remote displacement is applied to the other end. Since the deformation is significant, large deflection is enabled in the analysis setting. The calibrated data from Fig. 2 is imported to ANSYS for the simulation. The hyper-elastic model used is the Mooney–Rivlin 5 parameter model.

**Fig. 6:**
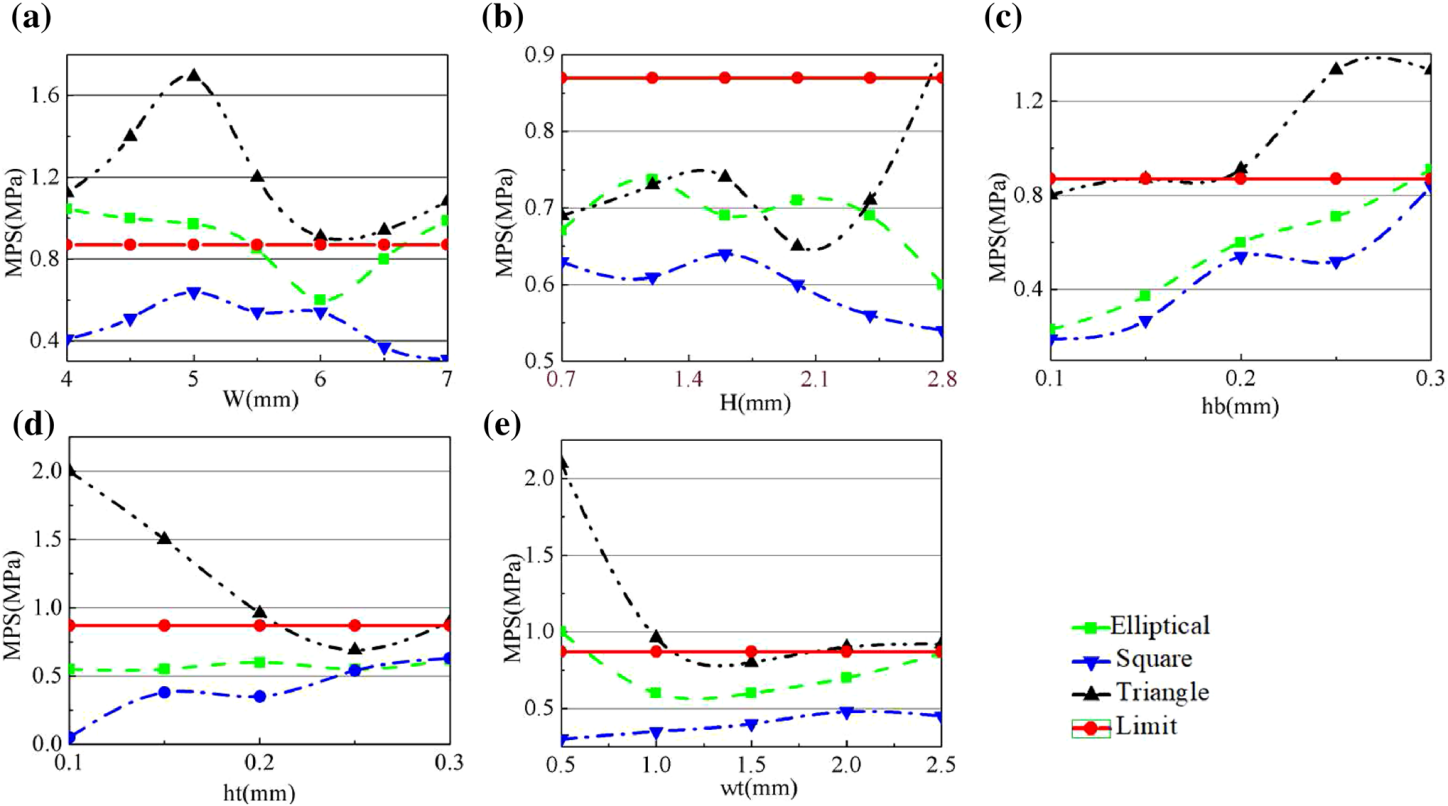
The maximum principal stress (MPS) during folding against different variables: **a** hinge width, *W*, **b** cavity height, *H*, **c** the bottom wall of cavity, *hb*, **d** the top wall of cavity, *ht*, and **c** the side walls of cavity, *wt*

**Fig. 7:**
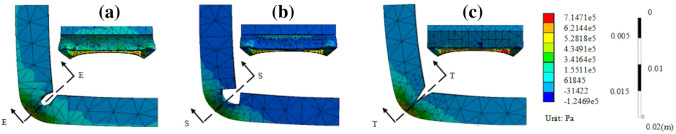
Simulation comparison of different hinges with parameters $W=6\,{\mathrm{mm}}, H=2.8\,{\mathrm{mm}}, ht=0.2\,{\mathrm{mm}}, hb=0.2\,{\mathrm{mm}}, T=5.5\,{\mathrm{mm}}$. **a** elliptical hinge and its section view. **b** square hinge and its section view. **c** triangle hinge and its section view

The failure of materials can be determined using the maximum stress criterion, which assumes that a material fails when the maximum principal stress (MPS) in a material element exceeds the material’s uniaxial tensile strength. Therefore, the MPS is reported here, and the material will fail if it exceeds 0.87 MPa (from Fig. 2). Different values for each of the five parameters are tested to obtain their contributions to the stress distribution. The MPS of the geometries are reported in Fig. 6. By comparing with the ‘Limit’ of 0.87 MPa, it can be seen that the ‘Triangle’ hinge often has higher stress than the ultimate stress, and although the ‘Elliptical’ hinge has some safe configurations, the choices are limited. Among them, the ‘Square’ hinge consistently has the lowest MPS and is below the limit in every case. The results are further verified by the visualization shown in Fig. 7, for a particular set of parameters. It reveals that the square hinge bends like a bar element with a lower curvature under the same folding compared to the elliptical and triangular hinges. From this, we can extrapolate that a sharper hinge geometry will result in a higher MPS. From Fig. 7a–c, the MPSs can be seen located below the cavity where the greatest elongation occurs. The values are 0.60 MPa, 0.54 MPa, and 0.91 MPa for the elliptical, square, and triangular hinges, respectively. All stresses are in the tensile direction.

It is concluded that the square hinge is the most suitable geometry for our purpose, and it is selected in this study. It allows a broader range of design parameters, which translates directly into a broader range of desired folding angles. The objective is to maximize the volume of the cavity and thus the moment $M_{in}$ which helps maintain the desired 3D shape. The opening of the hinge *W* is set as at most 6 mm and the top/bottom walls of the cavity are set as $ht = hb = 0.2\,{\mathrm{mm}}$, which are the maximum horizontal length and the minimum thickness of a support bridge that can be stably fabricated with the 3D printer being used. The depth of the hinge *d* is set based on the geometric assumption that the natural axis is located at the bottom of the opening (see Fig. 4). When the top of the hinge is closed by *W*, the hinge’s bottom will be elongated (*l*) according to the ratio between the heights. It should be smaller than the elongation at break ($\xi$), i.e.,

2 $$\begin{aligned} \frac{l}{W}=\frac{T-d}{d}\le \xi , \end{aligned}$$

where $\xi =1.4$. Given the 2D sheet’s thickness is $T=5.5\,{\mathrm{mm}}$, the depth of the hinge *d* needs to be greater than or equal to 2.3 mm. This paper uses $d=2.3\,{\mathrm{mm}}$. Thus, the height of the cavity *H* is at most 2.8 mm. Because *ht* and *hb* are dependent on *H*, and *wt* is constant, the two variables which show the highest affect on the cavity volume and selected for the control of the folding angle are *W* and *H*. The variables which are kept constant for the hinge designs have their values expressed in Table 2.

**Table 2:** Summary of the hinge variables

*T *(mm)	*d *(mm)	*ht *(mm)	*hb *(mm)	*wt *(mm)
5.5	2.3	0.2	0.2	1.5

### Modeling and design of folding angle

Hinge parameters (*W*, *H*) dictate the final folding angle of a hinge. Thus the inverse problem, which is more intuitive for design, selects a folding angle that dictates the parameters. The relationship between the parameters and the folding angle is obtained through a heuristic approach. Initially, we populate the design space through the forward method with predetermined values for *W* and *H*, obtain the resulting folding angles, and build a mathematical model to describe the relationship:

3 $$\begin{aligned} (W,H) = f(\alpha ) \end{aligned}$$

Before the design of experiments, a few trials have been made to identify the maximum folding angle. The largest and the most repeatable angle was achieved when $W=6\,{\mathrm{mm}}$, $H=2.8\,{\mathrm{mm}}$, resulting in a folding angle of $120^\circ$. Decreasing the value of *W* results in a direct decrease in the folding angle. However, there is a limit when *W* is less than 4 mm that results in interference in the hinge opening, which hinders folding. Therefore, we apply two variables to the control of the folding angle for two different instances. When the angle is large, it is controlled by the hinge width *W*, and the cavity height *H* is set to its maximum. When the angle is small and *W* cannot be further reduced, we modify *H* to control the angle, with *W* set to its minimum (i.e., 4 mm). Therefore, the experiments are designed based on *W* and *H*. *W* is ranging from 4 to 6 mm, and *H* is ranging from 0 to 2.8 mm, with a step size of 0.4 mm.

**Fig. 8:**
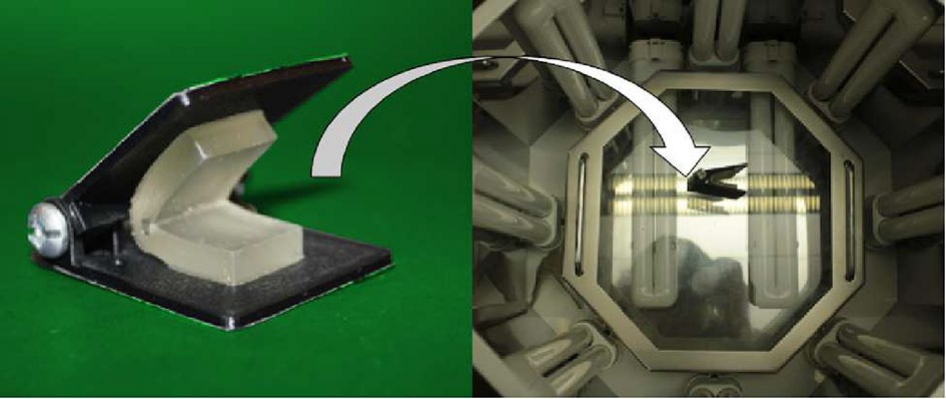
Folding device used for the calibration of folding angles and the post-curing process

All samples have a size of $40\,{\mathrm{mm}} \times 15\,{\mathrm{mm}} \times 5.5\,{\mathrm{mm}}$, and each configuration is repeated five times. They are fabricated based on the manufacturing process outlined in Sect. 3. The only difference is that before the post-curing step, the samples are folded with a simple folding device (see Fig. 8), and the assembly is put in a UV curing station. The device has two movable plates assembled by two screws that allow the adjustment of the opening angle. The final shape of a hinge is dependent on the balance between materials cured in different steps. The post-curing step requires holding the part at a given angle of $H_{\alpha}$ in order to set the shape of the uncured cavity resin. $H_{\alpha}$ could be considered as a design parameter to control the final angle. However, preliminary experiments demonstrated $\alpha$ was not sensitive to the holding angle. Additionally, as $H_{\alpha}$ is a process parameter, it would increase the complexity of manufacturing and assembly processes, which is not necessary to achieve the desired angles. For the sake of creating a unified process, we set the holding angle to $140^\circ$ in this calibration.

**Fig. 9:**
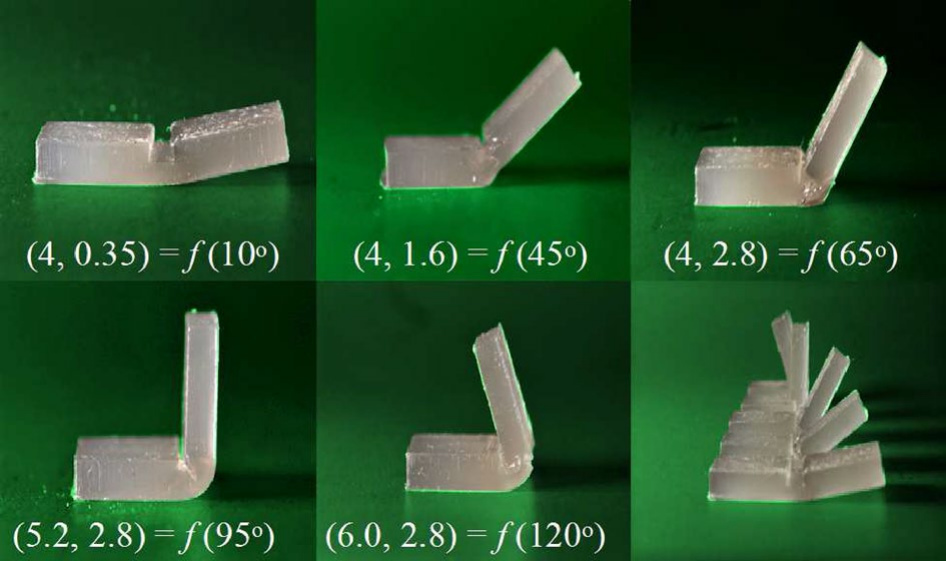
The bending angles $\alpha$ can be controlled by different values of (*W*, *H*)

**Fig. 10:**
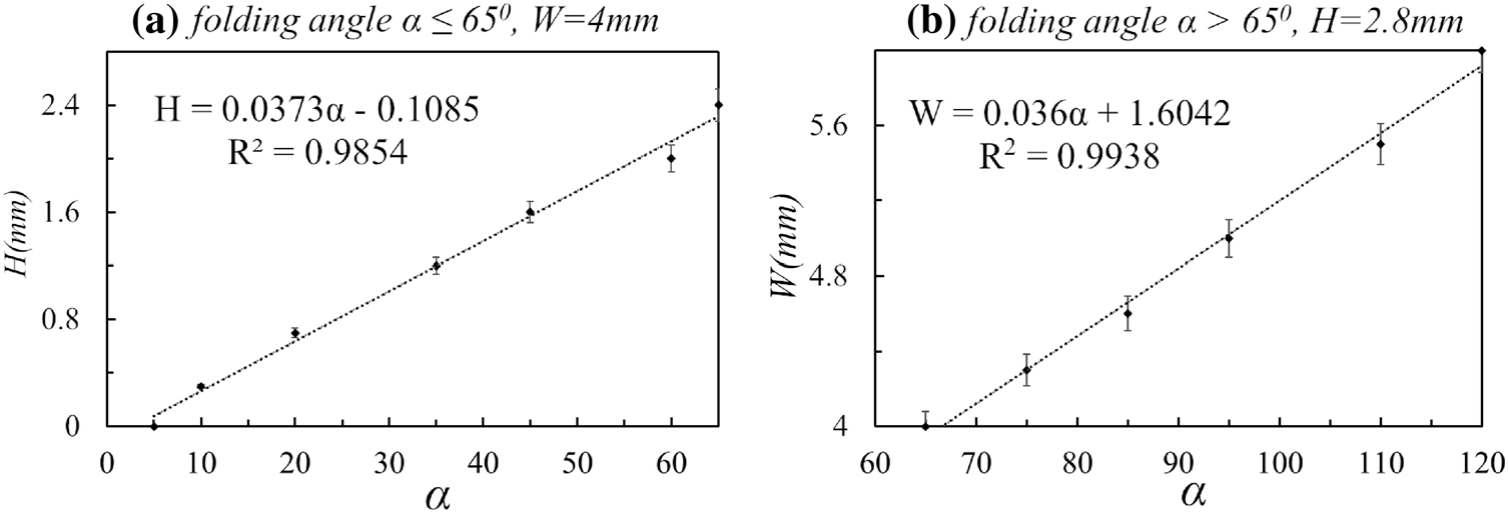
Linear regression of experimental data to calibrate the folding angle with the hinge parameters. **a** The cavity height (*H*) against small folding angle ($\alpha \le 65^\circ$). **b** The hinge width (*W*) against large folding angle ($65\le \alpha \le 120^\circ$). $R^2$ is the coefficient of determination

Some final shapes of the samples are shown in Fig. 9, and the experimental results (see “Appendix”) are plotted in Fig. 10 with two graphs: *H* against $\alpha$, and *W* against $\alpha$. Since the data sets are almost linear, a linear regression is used to model each relationship. The resultant $R^2$-value is 0.99. As a result, the empirical model to obtain the hinge parameters (*W* and *H*) for a given bending angle is defined as

4 $$\begin{aligned} (W,H) = f(\alpha ) = \left\{ \begin{array}{cc} (0.036 \alpha + 1.6042, \;\; 2.8) &{} \alpha >65^\circ \\ (4, \;\; 0.0373 \alpha -0.1085) &{} \alpha \le 65^\circ \\ \end{array} \right. \end{aligned}$$

This model’s exactness has been obtained for the material we are using; as such different resins will have to be calibrated and utilize their material properties for the parameter limits and method described.

### Interference between hinges

**Fig. 11:**
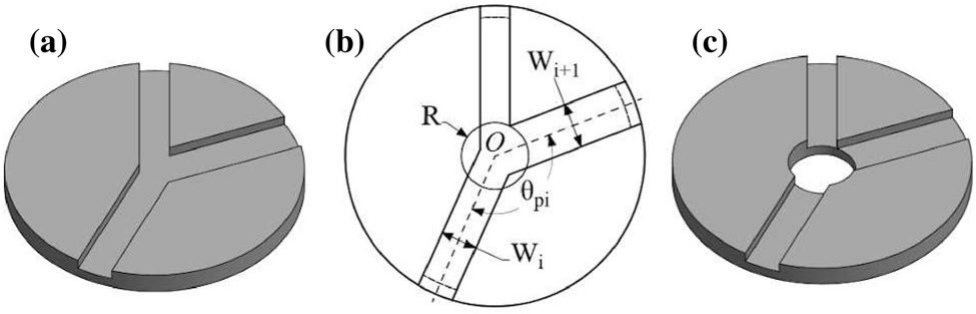
Interference of adjacent hinges. **a** 3D model when adjacent hinges meet up. **b** 2D sketch of adjacent hinges. **c** 3D model with residual hole

In an origami pattern, interference at a vertex may occur when multiple hinges are present. This causes interference and hinders the desired result, especially as the material’s thickness increases, seen in Fig. 11a. A common practice is introducing a hole at the vertex to decouple the hinges [27] to eliminate the interference. Referring to the 2*D* sketch Fig. 11b, the hole should be large enough such that the hinges do not have any overlap. Assume *i* and $i+1$ are two adjacent hinges with widths $W_i$ and $W_{i+1}$, respectively. The two hinges are intersecting at a vertex *O*, and the angle between the two hinges is $\theta _{pi}$. Because $W_i$ and $W_{i+1}$ are obtained from Sect. 4.2, it is possible to derive the minimum radius of residual hole in terms of $W_i$, $W_{i+1}$ and $\theta _{pi}$, applying the arc length formula:

5 $$\begin{aligned} R_i = (W_i + W_{i+1})/2\theta _{pi} \end{aligned}$$

If more than two hinges are intersecting at a vertex, the hole’s radius should be the maximum value among all the pairs of neighbouring hinges. Finally, Fig. 11c shows the modified 3D model.

## Experimental results

With the developed hinge design, fabrication method, and mathematical model, this section will apply them to test the methodology and demonstrate some possible usages. The same terminology in origami will be used to describe the patterns. For example, a hinge can be designed as a valley fold or a mountain fold. If it is a valley fold, the hinge’s opening is pointing upward with respect to the viewing direction. Otherwise, the opening points downward and is located at the back of the pattern. Unless otherwise stated, the thickness of samples in the experiments are designed as $T=5.5\,{\mathrm{mm}}$. Parts are manually folded and held by various holding mechanisms utilized on a case-by-case basis during the post-curing process, such as peg-boards, cable ties, and bar folders. For most examples illustrated below, a holding angle of $140^{\circ }$ was used during the post-curing process. Some intricate origami patterns cannot achieve said holding angle for every joint simultaneously; however, the experimental results and designed angles still closely correlate.

### Quasi-one-dimensional strips

**Fig. 12:**
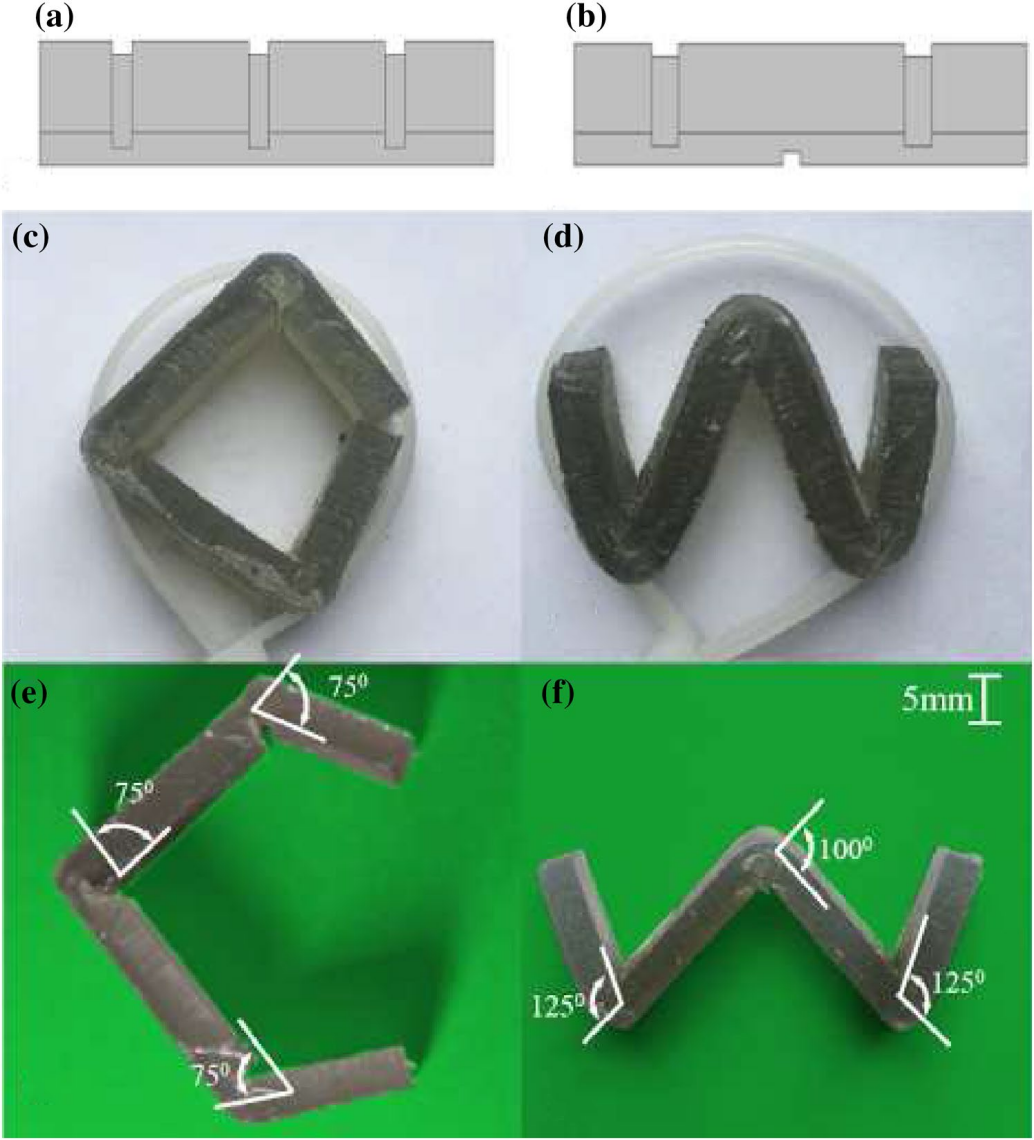
One-dimensional strips. **a** and **b** The 3D models of the letters ‘C’ and ‘W’. **c** and **d** The shape-setting is done by a cable tie. **e** and **f** The final shapes after the cable tie is removed

One-dimensional (1D) strips are relatively simple in topology but have many applications, such as robotics, mechanical spring, architectural design, or aesthetics [26]. They are used as the first example the verify the proposed method. Two letters—‘C’ and ‘W’—are selected for fabrication, where ‘C’ has folds in one direction, and ‘W’ has folds in both directions. Both are approximated by a 1D strip with three hinges. The CAD models of which are shown in Fig. 12a and b. The length and width of the strips are 100 mm and 15 mm, respectively. All three hinges of the letter ‘C’ are valley folds with a designed folding angle of $75^{\circ }$ (valley), which in turn give the hinge parameters $W=4.4\,{\mathrm{mm}}$ and $H=2.8\,{\mathrm{mm}}$. For the letter ‘W’, the folding angle of the middle hinge is designed as $100^{\circ }$ (mountain), and the angles of the side hinges are $120^{\circ }$ (valley). This gives the middle hinge opening downward, with the parameters $W=5.4\,{\mathrm{mm}}$ and $H=2.8\,{\mathrm{mm}}$, and the opening of side hinges upward, with the parameters $W=6.0\,{\mathrm{mm}}$ and $H=2.8\,{\mathrm{mm}}$.

Since the hinges are designed to facilitate folding in the desired direction, the shape-setting before post-curing can be as simple as using a cable tie to circle up the strips, and they are automatically formed into the shape of letters correspondingly, as shown in Fig. 12c and d. The actuation using a cable tie is the same for both models, and the diameter can be adjusted by stretching the rope. Although the holding angles for the hinges may not be the same under this overall actuation, the effect is negligible as long as they are larger than the desired angle, like discussed in Sect. 4.2. After the cable tie deforms the structures, the assembly is placed into the LC-3D Print Box for the post-curing. The final shapes obtained are shown in Fig. 12e and f. It can be seen that even after the rope is removed, the structures can stay in the deformed shapes on their own. All the hinges in the letter ‘C’ are maintained with an angle of around $75^{\circ }$ as designed. Similarly, the middle hinge of the letter ‘W’ is maintained at an angle of around $100^\circ$. There is a slight difference for the side hinges of ‘W’, and they have a final angle around $125^\circ$ (designed $120^\circ$), which might be caused by the manufacturing inaccuracy and the overstretching. We have also tried to press the structures flat, and they can still return to the configurations of ‘C’ and ‘W’.

### Pop-ups

**Fig. 13:**
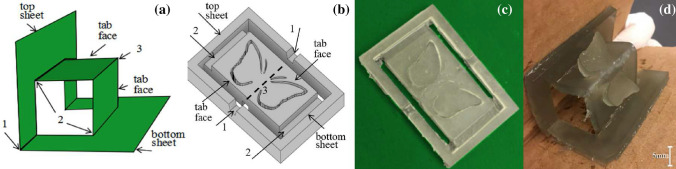
Pop-up. **a** The mechanism using parallel folds. **b** The CAD model of a pop-up design with a butterfly profile. **c** The printed 2D sheet. **d** A folder is used to fold the sheet and make the butterfly pop out

Pop-ups are the designs that arise from 2D configuration into 3D by simple folding like opening a page, and a common example is a pop-up book or card. Pop-ups come in different categories, depending on their geometry, including parallel folds, angle folds, and their combinations. For example, in parallel folds, the linkages are connected with the shape like a parallelogram (see Fig. 13a) and can be best represented as a four-bar linkage. By keeping the bottom sheet flat on the ground, moving the top sheet will change the angle of the middle hinge (labeled 1). Due to the parallelism, the opposite tabs will move accordingly and pop out from the original plane. This mechanism is employed with the proposed method to produce final 3D shapes with a simple folding method.

A profile of a butterfly is designed and engraved into the tabs, as shown in Fig. 13b. The full size of the model is $100\,{\mathrm{mm}} \times 60\,{\mathrm{mm}}$. A 5 mm gap is added between the outer frame and the tab to simulate cuts. There are five hinges on the sheet: two valley folds (labeled 1) at the middle of the outer frame, one mountain fold (labeled 3) in the center of the tab surface, and two more valley folds (labeled 2) connecting the frame and the tabs. All five hinges are designed to have a folding angle of $90^{\circ }$, which gives $W=5\,{\mathrm{mm}}$ and $H=2.8\,{\mathrm{mm}}$. The printed 2D sheet is shown in Fig. 13c, and it can be folded using a folding device similar to the one shown in Fig. 8. The folding and post-curing procedures are the same as those used to calibrate the folding angle with hinge parameters. It is worth noting that using this method—pop-up design with the help of a folding device, many different shapes can be fabricated with the same mechanisms by merely changing the middle pattern.

In this example, when the 2D sheet is actuated, all the hinges fold as designed and it forms a shape of a parallelogram as shown in Fig. 13d. While the tabs connected to the frame by hinges are bent, the engraved profile decouples the butterfly from the tabs, and thus the wings of the butterfly are free to pop out. In addition, since the degree to which the wings pop out from the tabs depends on the middle hinge angle, an interesting effect can be seen when the top sheet is moved back and forth—the wings are flapping similar to a flying butterfly. When the final shape is pressed flat, it can automatically return to the folded shape. This characteristic can facilitate packaging and transportation, giving a high packing ratio with 2D sheets and restoring to 3D shapes after unpacking.

### Action origami

**Fig. 14:**
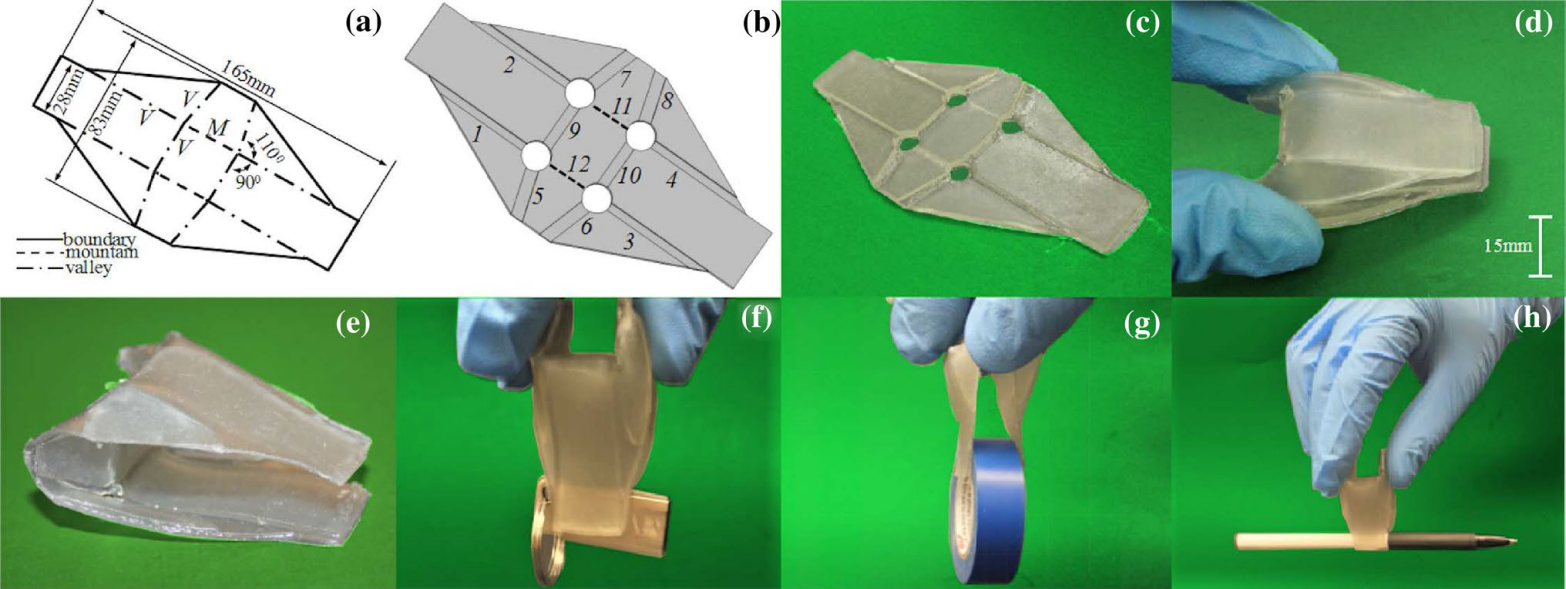
Oriceps. **a** The 2D pattern of oriceps [8]. **b** The CAD model with hinges designed. **c** The 2D polymerized sheet. **d** Shape-setting by actuation. **e** Final shape after post-curing. The oriceps is used to pick up **f** a USB key, **g** a roll of tape, and **h** a ball pen

Action origami is origami that can have actions after folding. One example is Oriceps [8], which is an origami-inspired forceps. Conventional forceps often require assembly from different parts to provide movements. However, product complexity makes the manufacturing process complicated, and the forceps challenging to clean. Due to these problems, Oriceps was proposed to be fabricated by a single sheet, and it can be actuated just like a standard forceps. This can get rid of the complicated assembly process, and it is potentially suitable for various scales from macro to micro. It has potential use as surgical forceps because it is compliant and can be easily sterilized.

To demonstrate that the proposed manufacturing method can also produce action origami, the Oriceps design [8] is employed in this paper (see Fig. 14a). The length and width of the model are 165 mm and 83 mm, respectively. There are in total twelve hinges (Fig. 14b): at the side, there are four long ones (1, 2, 3, 4) and four short ones (5, 6, 7, 8), all of which are valley fold; in the middle, there are four hinges, two of which are valley folds (9, 10) and two are mountain (11, 12). The long hinges (1, 2, 3, 4) are designed to have a folding angle of $40^{\circ }$. Thus the hinge parameters $W=4\,{\mathrm{mm}}$ and $H=1.6\,{\mathrm{mm}}$. The short hinges (5, 6, 7, 8) are of folding angle $120^{\circ }$ and hinge parameters $W=6.0\,{\mathrm{mm}}$ and $H=2.8\,{\mathrm{mm}}$. The middle two hinges (9, 10) that are valley folds have a folding angle of $90^{\circ }$—$W=5.0\,{\mathrm{mm}}$ and $H=2.8\,{\mathrm{mm}}$, and the last two mountain folds have an angle of $65^{\circ }$ with the hinge parameters $W=4.0\,{\mathrm{mm}}$ and $H=2.4\,{\mathrm{mm}}$. There are four vertices in the pattern, and they have the same configuration because of symmetry. The angles between hinges ($\theta _{pi}$) at a vertex are $90^{\circ }$, $110^{\circ }$, $70^{\circ }$ and $90^{\circ }$, respectively. Therefore, the diameters of the holes are calculated by Eq. (5), and the maximum interference results in a $4.09\,{\mathrm{mm}}$ diameter relief hole. Limited by the printing size of the 3D printer, the model cannot be fabricated in full scale, so it is uniformly scaled down by half for the physical test. This scaling may affect the shape-retention property and should be calibrated as well in the future. In this example, the effect is not significant, and the accuracy of folding angles on action origami can be lower. Since all the hinges are designed to bend in the desired direction—pre-folded, the same actuation to actuate the Oriceps can be applied to deform the 2D sheet to the shape-setting configuration, i.e., pulling from the back (see Fig. 14d). After post-curing, the final shape is kept in the 3D shape, as shown in Fig. 14e. It has also been tested by picking up different objects (Fig. 14f–h), and the results validate the functionality of the Oriceps fabricated by the proposed method. A video showing the Oriceps in action is available on YouTube: https://youtu.be/CTileFPnUAY.

### Bistable origami

**Fig. 15:**
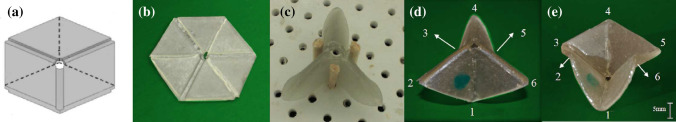
Waterbomb base. **a** The CAD model—dotted lines indicate the hinges with mountain folds. **b** The 2D polymerized sheet after 3D printing. **c** Shape-setting configuration for post-curing. **d** One stable state with the vertex above the flat plane. **e** The other stable state with the vertex below the flat plane

Bistable origami is a structure that has two stable states while maintaining the configuration of folds the same, i.e., a valley fold is still a valley fold, and vice versa. It can serve as a mechanical memory unit that snaps between two stable states of the folded structure under external stimulus with applications in electronics acting as a switch. One classic bistable origami is the waterbomb base [7], which has only one vertex intersected by six hinges with alternating mountain and valley folds, as shown in Fig. 15a It is used to test if the bistable property can be replicated by the proposed method.

The 2D model is designed in a hexagon shape, and the length of its sides is 55 mm. Among the six hinges, three are valley folds, and three are mountain folds. The valley folds have a folding angle of $75^{\circ }$, which gives the hinge parameters $W=4.4\,{\mathrm{mm}}$ and $H=2.8\,{\mathrm{mm}}$, and the mountain folds have an angle of $30^{\circ }$, thus $W=4.0\,{\mathrm{mm}}$ and $H=1.1\,{\mathrm{mm}}$. The diameter of the hole at the vertex is calculated as $6\,{\mathrm{mm}}$. Due to the limit of print size, the model is scaled down to around half. The printed 2D sheet is shown in Fig. 15b. In this example, a simple fixture is used to set the shape, as shown in Fig. 15c. The final 3D shape is shown in Fig. 15d, which is one of the two stable states. The other one can be obtained by pushing the vertex in the center down to make the structure flat, and after passing the flat state, the structure will snap through to the resultant shape, as shown in Fig. 15e. It can be seen that all the hinges still have the same valley/mountain configuration. This example demonstrates that the part fabricated by the proposed method indeed has the characteristics of bistable origami. Thus it is promising to apply the method for other more complex origami patterns.

## Conclusion and future work

In the paper, a new manufacturing method based on DLP technology is developed to fabricate origami-based components. The objective is to leave parts of the model uncured during the 3D printing step and then cure them in the post-processing step to set the final shape. To test the capability of this method, finite element analysis in ANSYS is conducted to find the most suitable hinge geometry. Due to the relatively lower maximum principal stress, the square hinge is chosen to be modified for our objective. A cavity is incorporated into the hinge design, the goal of which is to trap the uncured resin within the part. A mathematical model is also established to describe the relationship between the hinge parameters (hinge width and cavity height) and the final folding angle. The model is applied to fabricate a few examples with the given final shape to validate the method. Firstly, the one-dimensional strips demonstrate that the method can fabricate multiple hinges simultaneously, even if they have different target angles. Secondly, the pop-up butterfly shows that intricate patterns and geometry can be fabricated with the same folding mechanism by utilizing the pop-up concept. Thirdly, an origami-based forceps is printed to prove that the method can produce action origami, which can function after folding. Finally, a bistable waterbomb base is fabricated to reveal that the printed part shares origami characteristics. Thus it is promising to apply the method to other existing origami patterns.

Although the results are encouraging, there are some limitations of the method. One limitation is that the maximum folding angle that can currently be achieved is only $120^{\circ }$, which is not enough to produce sharper features. In the future, we will explore other structure and hinge designs to increase the maximum folding angle. However, since the holding angle needs to be larger than the folding angle, it is not quite possible to fabricate extremely sharp features, like $180^\circ$ angles. In such cases, other self-locking features may need to be included. Another challenge is the need for a holding mechanism during the post-curing process. Currently, a manual holding process is performed, and thus the moment applied on each hinge might be different, which means that some hinges may experience over-folding while the others are still under-folding. However, future work could unify this process and make the holding mechanism part of the design. Additionally, it may be possible to develop origami that folds using a single actuation mechanism, limiting the need for holding devices. We will investigate the moment at each hinge and modify the hinges, if needed, to realize simultaneous folding. Finally, the folding angle’s effect should be further investigated and potentially added as a design parameter. In essence, the holding mechanism would try to maximize the holding angle. That angle would be used as an input for the hinge’s geometry, thus eliminating the process aspect and maintaining a geometry methodology.

## Appendix: calibration data

Here listed the test data for calibrating the mathematical model (Eq. 4) derived in this paper. They are the average values of the five repeated samples (Tables 3 and 4).

**Table 3:** Folding test data based on different (*H*, $\alpha$)

$ht\,{\mathrm{(mm)}}$	$W\,{\mathrm{(mm)}}$	$H\,{\mathrm{(mm)}}$	$H_{\alpha}$ ($^\circ$)	$\alpha$ ($^\circ$)
0.2	4	0	140	5
0.2	4	0.35	140	10
0.2	4	0.7	140	20
0.2	4	1.2	140	35
0.2	4	1.6	140	45
0.2	4	2.4	140	60
0.2	4	2.8	140	65

**Table 4:** Folding test data based on different (*W*, $\alpha$)

*ht* (mm)	*hb* (mm)	*H* (mm)	*W* (mm)	$H_{\alpha}$ ($^\circ$)	$\alpha$ ($^\circ$)
0.2	0.2	2.8	4	140	65
0.2	0.2	2.8	4.4	140	75
0.2	0.2	2.8	4.8	140	85
0.2	0.2	2.8	5.2	140	95
0.2	0.2	2.8	5.6	140	110
0.2	0.2	2.8	6.0	140	120
